# Salivary Proteomic Signatures in Pregnant Women With Excessive Gingival Bleeding

**DOI:** 10.1111/odi.70127

**Published:** 2025-11-02

**Authors:** Gerson Aparecido Foratori‐Junior, Clovis Bergamin Griso, Amanda Borges Pirondi, Laura Teodoro de Marchi, Talita Mendes Oliveira Ventura, Larissa Tercilia Grizzo, Roosevelt da Silva Bastos, Alejandra Chaparro, Nagihan Bostanci, Marília Afonso Rabelo Buzalaf

**Affiliations:** ^1^ Department of Biological Sciences, Bauru School of Dentistry University of São Paulo Bauru São Paulo Brazil; ^2^ Department of Pediatric Dentistry, Orthodontics, and Public Health, Bauru School of Dentistry University of São Paulo Bauru São Paulo Brazil; ^3^ Department of Periodontology, Centre for Biomedical Research, Faculty of Dentistry Universidad de los Andes Santiago Chile; ^4^ Division of Oral Health and Periodontology, Department of Dental Medicine Karolinska Institutet Stockholm Sweden

**Keywords:** gingivitis, oral health, pregnancy, proteomics, saliva

## Abstract

**Objective:**

This observational, cross‐sectional, and analytical study aimed to explore the pathophysiological mechanisms of gingivitis in pregnancy by analyzing the salivary proteomic profile according to gingival bleeding status.

**Materials and Methods:**

Pregnant women at the 27th week of gestation or beyond were categorized into two groups: those with excessive gingival bleeding on probing (BOP > 50%; G1) and those without generalized gingivitis (BOP < 30%; G2). A comprehensive full‐mouth periodontal examination was performed. Unstimulated whole‐mouth saliva samples were collected and individually processed using Nano Liquid Chromatography Electron Spray Ionization Tandem Mass Spectrometry (nLC‐ESI‐MS/MS).

**Results:**

Proteomic analysis identified 187 salivary proteins, with 75 shared between groups. Sixty proteins were upregulated and seven were downregulated in G1. The most upregulated were Protein *S100‐A9* (16‐fold), *Neutrophil Defensins 1* and *3* (7‐fold), *Protein S100‐A8* (5‐fold), *Beta‐2‐Microglobulin* (4‐fold), and multiple immunoglobulin isoforms. *Histatin‐3* was the only protein downregulated by more than 2‐fold. Gene Ontology analysis revealed significant enrichment in processes related to antimicrobial humoral response, bacterial defense mechanisms, and immune regulation, reflecting the inflammatory state.

**Conclusion:**

These findings provide insights into the salivary proteomic alterations associated with generalized gingivitis in pregnancy, particularly highlighting immune and antimicrobial pathways linked to excessive gingival inflammation.

## 1 Introduction

During pregnancy, significant physiological changes occur to support maternal adaptation and fetal development. These changes are evident across all three trimesters, with pronounced effects during the second and, especially, the third trimester due to elevated levels of estrogen and progesterone. These hormonal fluctuations induce systemic alterations, including notable impacts on the oral cavity (Sachelarie et al. 2024). The etiology of oral manifestations during pregnancy is primarily associated with hormonal changes, which increase vascular dilation in the gingival area (Pecci‐Lloret et al. 2024). Additionally, reduced antimicrobial activity of peripheral neutrophils, along with imbalances in immune regulators and antioxidants/reactive oxygen species, contribute to the higher prevalence of periodontal disease during pregnancy (Fuhler 2020; Meriç et al. 2023).

Periodontal diseases include: (a) gingivitis, characterized by inflammation of the gingiva without loss of connective tissue attachment, which may occur in an intact or reduced periodontium (e.g., stable periodontitis or gingival recession without periodontitis); and (b) periodontitis, defined in the 2018 classification as a chronic multifactorial inflammatory disease associated with dysbiotic dental biofilms and characterized by progressive destruction of the tooth‐supporting apparatus. Periodontitis manifests as clinical attachment loss, radiographic bone loss, periodontal pocketing, and gingival inflammation and is further staged and graded according to severity, extent, and risk factors (Caton et al. 2018; Raju and Berens 2021). Gingivitis is the most common oral disease during pregnancy and can reach a prevalence of over 86% (Chen et al. 2022). This is a biofilm‐dependent disease; thus, gestation itself does not cause gingivitis. However, inadequate or absent oral hygiene, combined with vascular, hormonal, and immune changes during pregnancy, can increase the predisposition to gingivitis and exacerbate existing cases (Armitage 2013; Gil‐Montoya et al. 2023; Pecci‐Lloret et al. 2024).

With technological advancements, numerous studies have focused on identifying potential disease biomarkers in body fluids using proteomic approaches, particularly Mass Spectrometry, due to its exceptional sensitivity. Saliva proteomic analysis has emerged as a significant area of scientific research, offering strong potential for disease diagnosis through a non‐invasive and easily accessible method (Ahmad et al. 2024).

Recent studies have explored salivary proteomics to uncover biomarkers associated with gingivitis during pregnancy. (Balan et al. 2021) utilized isobaric tags for relative and absolute quantification (iTRAQ) and demonstrated that pregnant women with gingivitis exhibit enhanced neutrophil‐mediated immune responses and antioxidant defense mechanisms. Additionally, the study identified reduced levels of salivary cystatins and antimicrobial proteins as potential predisposing factors for gingivitis in healthy pregnant women, with *Cystatin‐C* significantly lower in those with the condition (Balan et al. 2021). Complementary findings by (Hassan et al. 2018), using enzyme‐linked immunosorbent assays (ELISA), suggested that elevated salivary levels of annexin‐1 could serve as a specific biomarker for gingivitis in pregnancy, supporting its use in early non‐invasive screening (Hassan et al. 2018).

Nevertheless, these previous studies primarily evaluated pregnant women with general gingivitis, without specifically distinguishing clinical subgroups based on bleeding severity. To the best of our knowledge, no study has yet used Mass Spectrometry to characterize the salivary proteomic profile of pregnant women stratified by excessive gingival bleeding (i.e., bleeding on probing ≥ 50%). Our study addresses this gap by exploring distinctive protein signatures, such as members of the S100 family (*S100‐A8/A9*) and cysteine protease inhibitors (*Cystatin‐C*), that may underlie exaggerated inflammatory and immune responses in severe forms of pregnancy gingivitis.

This study therefore aimed to investigate the pathophysiological mechanisms of gingivitis during the third trimester of pregnancy by analyzing the salivary proteomic profile of pregnant women, with and without excessive gingival bleeding on probing, recruited from Primary Health Care units in Brazil. We hypothesized that pregnant women with increased gingival bleeding would present a distinct proteomic signature, reflecting heightened inflammatory and immune activity, which could help to explain the greater severity of gingivitis observed in this group.

## 2 Material and Methods

This observational, cross‐sectional, and analytical study was conducted in accordance with *Strengthening the Reporting of Observational Studies in Epidemiology* (STROBE) (von Elm et al. 2007) and was registered in the ReBEC (https://ensaiosclinicos.gov.br/rg/RBR‐9mtvmqj) (registered on 13 August 2024) under the protocol number RBR‐9mtvmqj and the Universal Trial Number (UTN): U1111‐1309‐1168.

### 2.1 Ethics Statement

In compliance with the protocol established by the Declaration of Helsinki (released 1975, and revised in 2013), this study was approved by a Research Ethics Committee from Bauru School of Dentistry, University of São Paulo (CAAE 77660724.3.0000.5417). Participants were included in the study only after providing written consent.

### 2.2 Participants Recruitment

Participants were consecutively recruited from Primary Health Care units in Bauru, São Paulo, Brazil, between March and July 2024. Inclusion criteria included pregnant women in the third trimester of pregnancy (from the 27th gestational week), aged between 18 and 40 years, with regular follow‐up by an obstetrician, and adequate cognitive and neuromotor abilities to ensure comprehension of the study and the capacity to perform regular oral hygiene. Pregnant women were paired by age and socioeconomic level (schooling level and household monthly income) to ensure that the sample is homogeneous, minimizing bias in the proteomic profile analysis. Exclusion criteria comprised a diagnosis of periodontitis based on periodontal parameters; systemic arterial hypertension (blood pressure ≥ 140/90 mmHg) diagnosed before or during pregnancy; preeclampsia; gestational diabetes mellitus (blood glucose levels of 92–125 mg/dL fasting, ≥ 180 mg/dL after 1 h, or 153–199 mg/dL after 2 h); malnutrition (pre‐gestational BMI < 18.50 kg/m^2^); obesity (pre‐gestational BMI ≥ 30.00 kg/m^2^); diagnosis or suspicion of SARS‐CoV‐2 infection; medical recommendation for absolute rest; diagnosis of other systemic diseases; use of antibiotics or medications that could interfere with periodontal condition or salivary flow during pregnancy; hyposalivation (flow < 0.25 mL/min); multiple tooth loss (more than two teeth per hemiarch); tooth loss prior to pregnancy due to periodontitis; ongoing orthodontic or periodontal treatment; previous surgical periodontal treatment; and use of alcohol, tobacco, or illicit drugs during pregnancy.

For sample grouping, full‐mouth periodontal parameters, including probing depth (PD) and clinical attachment level (CAL), were recorded by a calibrated examiner (kappa = 0.95; ICC= 0.88) using a standard clinical periodontal probe (Hu‐Friedy, Frankfurt, Germany). Only women without any signs of bone loss (all sites with CAL ≤ 3 mm) were included in the study. Participants were diagnosed with gingivitis based on the parameter of bleeding on probing (BOP), as described by (Trombelli et al. 2018). In line with that framework, generalized gingivitis is defined as BOP ≥ 30%. For the purposes of this study, however, we excluded the intermediate range of 30%–50% to achieve a clearer biological distinction between groups. This decision was made to reduce the risk that participants with borderline values (e.g., 29% vs. 31%) would present overlapping proteomic profiles, which could obscure meaningful differences. Thus, women with BOP ≥ 50% were allocated to G1 (exposure group), while those with BOP ≤ 30% were allocated to G2 (control group). BOP was assessed dichotomously (present/absent) at six sites per tooth (three buccal and three palatal/lingual), following the standardized and validated index proposed by (Ainamo and Bay 1975). The final sample therefore comprised two groups of pregnant women: with excessive gingival bleeding on probing (BOP ≥ 50%; G1 = 9) and without generalized gingivitis (BOP ≤ 30%; G2 = 9). The sample size adopted in this study is in accordance with the literature, based on previous proteomic analysis protocols (Balan et al. 2021, Foratori‐Junior et al. 2022, Foratori‐Junior et al. 2023, Marchi et al. 2025, Ventura et al. 2018, Ventura et al. 2022).

### 2.3 Saliva Sample Collection

Unstimulated whole‐mouth saliva samples were collected following previous protocol (Gümüş et al. 2015). Participants were instructed to remain seated, upright, and at rest for 15 min before collection. They first rinsed their mouths with 5 mL of deionized water and expelled the contents. Unstimulated saliva was then collected for 10 min into a sterile 50 mL plastic Falcon tube kept on ice. The total saliva volume collected during this period was recorded. Immediately after collection, saliva samples were centrifuged at 4500 g for 15 min at 4°C to remove debris (Foratori‐Junior et al. 2022; Foratori‐Junior et al. 2023; Marchi et al. 2025; Ventura et al. 2018; Ventura et al. 2022). The resulting supernatant was carefully collected and stored at −80°C until proteomic analysis.

### 2.4 Preparation of Saliva Samples for Proteomic Analysis

Proteomic analysis was performed based on a previously described protocol (Ventura et al. 2018). In summary, saliva supernatant samples were analyzed individually using mass spectrometry, and the sample preparation followed 7 steps: (1) Extraction; (2) Concentration; (3) Quantification; (4) Reduction and Alkylation; (5) Digestion; (6) Desalinization and Purification; and (7) Resuspension.

For protein extraction, 1000 μL aliquots of the supernatant from unstimulated whole‐mouth saliva from each participant were mixed with 1000 μL of an extraction solution containing 6 M urea and 2 M thiourea in 50 mM NH_4_HCO_3_ (pH 7.8). Samples were vortexed for 10 min at 4°C, sonicated for 5 min, and centrifuged at 20,817 *g* for 10 min at 4°C. This three‐step cycle was repeated three times. The samples were then concentrated using Amicon tubes (Amicon Ultra‐15 Centrifugal Filter Units—Merck Millipore, Tullagreen, County Cork, Ireland) to a final volume of approximately 150 μL. To quantify total protein, a 1 μL aliquot from each sample was analyzed using a colorimetric method (Bradford) (Bio‐Rad, Hercules, California, USA), and 100 μg/protein was standardized for each sample prior to digestion for the differential expression analysis.

Proteins were reduced with 5 mM dithiothreitol for 40 min at 37°C and alkylated with 10 mM iodoacetamide for 30 min in the dark. Enzymatic digestion was then performed by incubating the samples with 2% trypsin (w/w) (Thermo Scientific Pierce Trypsin Protease, Rockford, Illinois, USA) for 14 h at 37°C. Digestion was stopped by adding 10 μL of 5% trifluoroacetic acid. Afterwards, samples were desalted and purified using C18 Spin columns (Thermo Scientific, Rockford, Illinois, USA). After drying, samples were subsequently resuspended in a solution containing 3% acetonitrile and 0.1% formic acid and subjected to mass spectrometry analysis (nanoLC‐ESI‐MS/MS) (Waters, Manchester, New Hampshire, UK) (Ventura et al. 2018).

### 2.5 Label‐Free LC‐ESI‐MS/MS Shotgun Proteomics

Peptide identification was conducted using a Xevo G2 QTof mass spectrometer connected to a nanoACQUITY system (Waters Co., Manchester, New Hampshire, USA), operating in positive ion nanoelectrospray mode. Data acquisition was performed using the MSE method with high‐energy scans (19–45 V), enabling simultaneous collection of precursor and fragment ion data in a single injection. The acquisition scan range spanned from 50 to 2000 Da. A fibrinopeptide [Glu1] solution (1 pmol/μL), delivered at a flow rate of 0.5 μL/min, served as the fixative spray to ensure reproducibility and accuracy (Ventura et al. 2018).

The acquired LC‐MSE data were processed and analyzed using ProteinLynx GlobalServer (PLGS) software version 3.0.3 (Waters Co., Manchester, UK). Proteins were identified through the software's ion counting algorithm by comparing the spectra against the *Homo sapiens* reviewed database from UniProt (UniProtKB/Swiss‐Prot, available at http://www.uniprot.org/). Each protein was examined based on its respective accession number, while redundant sequences, reverse matches, and fragments were excluded from analysis. Only proteins identified with a confidence level exceeding 95% were included in the quantitative analysis. The False Discovery Rate (FDR) value was applied in our results, and it was equal to 4.

For protein quantification, PLGS employed Monte Carlo algorithms, which use pseudo‐random numbers to test potential solutions and calculate the likelihood of a protein's presence based on Bayes' theorem. Internal normalization of results was performed using the Waters MassPREP Yeast Enolase Digestion Standard (SKU 186002325) to account for the total number of protein sequences analyzed. This standard was added to the samples during the resuspension step, following preparation and prior to submission to the mass spectrometer. Proteins exhibiting upregulation were reported with confidence as 1 ‐ p > 0.95, while downregulated proteins were characterized by *p*‐values < 0.05 (Ventura et al. 2018). Differences in protein expression between groups were statistically evaluated using the *t*‐test, with significance set at *p* < 0.05.

### 2.6 Protein Validation by MILLIPLEX Multiplex Immunoassay

The protocol recommended by the manufacturer for the MILLIPLEX Human Kidney Injury Magnetic Bead Panel 6 (MilliporeSigma, Burlington, MA, USA) was adopted for the validation of *β‐2‐Microglobulin* and *Cystatin‐C* in saliva samples. Initially, a mixture of recombinant proteins was prepared to generate the standard curve. The wells of the 96‐well plate were pre‐wetted with wash buffer, aspirated, and dried. Patient samples, together with standards and quality controls, were added in duplicate. Subsequently, magnetic microspheres coated with capture antibodies specific for the analytes were added, the plate was sealed, protected from light, and incubated overnight (18 h) at 4°C under agitation (600 rpm). After incubation, the wells were washed with wash buffer, and biotinylated detection antibodies were added, followed by incubation for 1 h at room temperature (25°C), protected from light. Then, streptavidin‐phycoerythrin conjugate was added to all wells and incubated for 30 min at room temperature (25°C) under gentle agitation (600 rpm). After final washes, the beads were resuspended in sheath fluid and shaken (600 rpm) at room temperature (25°C) for 5 min. Data acquisition was performed on the MAGPIX instrument (Luminex Corp.; Austin, TX, USA) using xPONENT software, with a minimum of 50 beads per analyte collected. Data analysis was performed with MILLIPLEX Analyst 5.1 software (MilliporeSigma, Burlington, MA, USA), and concentrations were calculated based on the standard curve.

### 2.7 Statistical Analyses and Bioinformatics for Proteomics

Although the sample size of this study was guided by previous in vivo salivary proteomic investigations, we also conducted a *post hoc* power analysis using G*Power 3.1, considering salivary levels of *β‐2‐Microglobulin* and *Cystatin‐C* obtained in the validation step as outcomes. It is important to emphasize that sample size calculations should primarily rely on predefined primary outcomes. However, because this is an exploratory study designed to identify multiple differentially expressed salivary proteins, no single parameter could be established a priori to guide the calculation. In this context, the validation step provides supportive evidence for the adequacy of our sample size. Using α = 0.05, Cohen's d effect sizes were 1.33 for *β‐2‐Microglobulin* and 1.23 for *Cystatin‐C*, resulting in achieved powers of 85.4% and 80.7%, respectively.

Statistical analyses were conducted using IBM SPSS Statistics (version 25.0, IBM Corp, Armonk, NY, USA). The normality of the variables was assessed using the Shapiro–Wilk test, while variance homogeneity was verified through Levene's test. For comparisons of quantitative variables with a normal distribution between groups, the *t*‐test was applied. In cases where variables did not meet the normality assumption or for ordinal qualitative variables, the Mann–Whitney test was utilized.

Protein categories were based on the Gene Ontology (GO) regarding biological processes, molecular functions, immune systems, and cellular components, using ClueGo plugins of the Cytoscape 3.10.3 software. Functional distribution of proteins identified with differential expression (up‐ and down‐regulated in G1) was performed in the comparison between groups. The terms of significance (κ = 0.04) and distribution were based on the percentage of the number of associated genes. STRING platform (https://string‐db.org) was used to determine protein–protein interactions between groups (only up‐ and down‐regulated in G1 with fold change greater than 2). Mass spectrometry proteomic data have been deposited to the ProteomeXchange Consortium via the PRIDE partner repository with the dataset identifier PXD059154.

## 3 Results

Participants were matched for age and socioeconomic status, with a mean sample age of 26.9 years (±7.09). The groups showed no significant differences in anthropometric parameters, including pre‐gestational BMI, gestational BMI, and gestational weight gain, confirming the homogeneity of the sample and minimizing potential biases in the salivary proteomic analysis (Table 1). As expected, G1 exhibited a significantly higher percentage of surfaces with visible biofilm (*p* = 0.003) and bleeding on probing (BOP) (*p* < 0.001), which served as the grouping variable. G1 also demonstrated slightly higher probing depth (PD, *p* = 0.029) and clinical attachment level (CAL, *p* = 0.040); however, these differences were clinically irrelevant, as participants with CAL > 3 mm were excluded. Additionally, no significant differences were observed between the groups in terms of salivary flow rate or total salivary protein quantification (Table 1).

**TABLE 1:** Summary of Contextual, Oral, and Salivary Features of the Sample.

	G1 (*n* = 9) mean ± SD median [1st‐3rd quartiles]	G2 (*n* = 9) mean ± SD median [1st‐3rd quartiles]	*P*
Age (years)	26.9 ± 7.17	26.9 ± 7.01	1.000*
Gestational Weight Gain (kg)	10.6 ± 6.65	10.8 ± 4.13	0.891*
Pre‐pregnancy BMI (kg/m^2^)	24.40 [22.70–24.80]	22.50 [22.30–24.10]	0.222^a^
Pregnancy BMI (kg/m^2^)	28.80 [26.80–29.40]	26.00 [25.40–28.10]	0.258^a^
Daily toothbrushing	2 [2–3]	3 [3–3]	0.175^a^
Daily flossing	0 [0–1]	0 [0–1]	0.915^a^
BOP (%)	58.30 [50.00–69.90]	26.80 [23.80–29.60]	< 0.001^a^
Biofilm (%)	78.60 ± 17.40	50.10 ± 17.20	0.003*
PD (mm)	2.20 ± 0.20	2.02 ± 0.09	0.029*
CAL (mm)	2.20 ± 0.20	2.04 ± 0.08	0.040*
Salivary flow (mL/min)	0.62 ± 0.05	0.61 ± 0.04	0.476*
Total protein quantification (ug/ptn)	51.8 [32.9–54.5]	74.7 [52.2–101]	0.157^a^

Abbreviations: BMI, body mass index; CAL, clinical attachment level; G1, with excessive Bleeding on Probing (BOP > 50%); G2, without generalized gingivitis (BOP < 30%); P, significance level; PD, probing depth; SD, standard deviation.

* *t*‐test.

^a^ Mann–Whitney.

**FIGURE 1:**
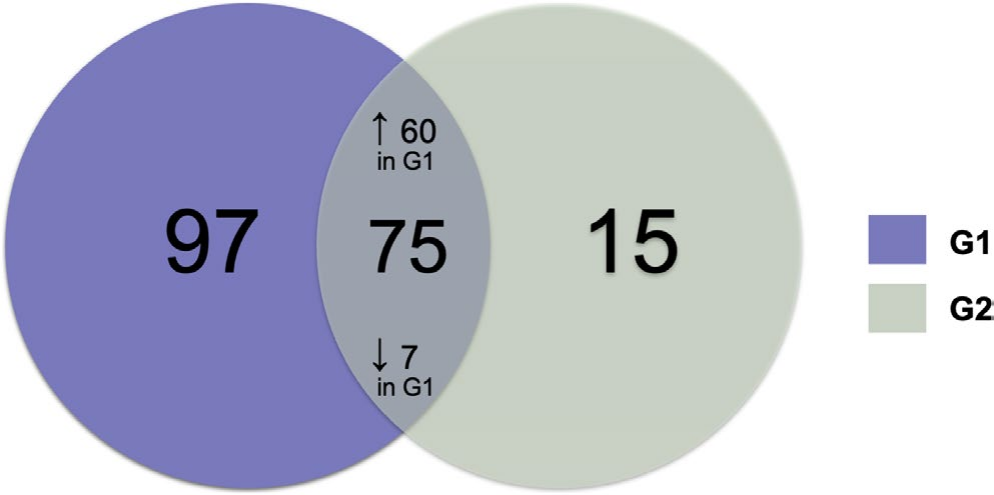
Venn diagram showing the quantitative distribution of proteins between groups.

Proteomic analysis revealed a total of 187 proteins, of which 75 were common between the two groups, with 60 proteins being up‐regulated in G1 and 7 being down‐regulated in G1 (Figure 1). The complete list of proteins identified in the proteomic analysis can be found in Supplementary File 1.

Among the 60 upregulated proteins in G1, 42 exhibited fold change (FC) values greater than 2. The most prominent proteins included: *Protein S100‐A9* (FC > 16), *Neutrophil Defensins 1* and *3* (FC > 7), *Protein S100‐A8* (FC > 5), *Beta‐2‐Microglobulin* (FC > 4), and several immunoglobulin isoforms, such as *Immunoglobulin Heavy Constant Mu*, *Immunoglobulin Kappa Constant*, *Immunoglobulin Kappa Light Chain*, *Immunoglobulin Mu Heavy Chain*, and *Immunoglobulin Gamma‐1 Heavy Chain*, among others. Among the 7 downregulated proteins in G1, only *Histatin‐3* showed FC greater than 2 (FC = 3.03), but other relevant proteins were *Alpha‐1‐antitrypsin*, *Cystatin‐S* and *‐SN* (Table 2).

**TABLE 2:** Differentially Expressed Proteins Between Groups (Up‐ and Down‐Regulated in G1).

Accession number	Protein name	Gene	Score	Fold Change	Log (e)	SD	*P*	ED
P06702	*Protein S100‐A9*	*S100A9*	237	16.44	2.80	0.02	< 0.01	↑
P59665	*Neutrophil defensin 1*	*DEFA1; DEFA1B*	489	7.46	2.01	0.07	< 0.01	↑
P59666	*Neutrophil defensin 3*	*DEFA3*	489	7.39	2.00	0.07	< 0.01	↑
P52209	*6‐phosphogluconate dehydrogenase, decarboxylating*	*PGD*	46	5.53	1.71	0.17	< 0.01	↑
P01871	*Immunoglobulin heavy constant mu*	*IGHM*	112	5.31	1.67	0.08	< 0.01	↑
P05109	*Protein S100‐A8*	*S100A8*	1319	5.05	1.62	0.07	< 0.01	↑
Q5VSP4	*Putative lipocalin 1‐like protein 1*	*LCN1P1*	319	4.90	1.59	0.08	< 0.01	↑
P01834	*Immunoglobulin kappa constant*	*IGKC*	626	4.81	1.57	0.03	< 0.01	↑
P0DOX7	*Immunoglobulin kappa light chain*	*IGK*	626	4.71	1.55	0.1	< 0.01	↑
P61769	*Beta‐2‐microglobulin*	*B2M*	182	4.48	1.50	0.09	0.01	↑
P0DOX6	*Immunoglobulin mu heavy chain*	*IGM*	96	4.31	1.46	0.15	< 0.01	↑
P0DOX5	*Immunoglobulin gamma‐1 heavy chain*	*IGG1*	83	3.94	1.37	0.07	< 0.01	↑
Q9BYX7	*Putative beta‐Actin‐like protein 3*	*POTEKP*	94	3.60	1.28	0.08	< 0.01	↑
P09211	*Glutathione S‐transferase P*	*GSTP1*	72	3.46	1.24	0.16	< 0.01	↑
P00738	*Haptoglobin*	*HP*	77	3.32	1.20	0.08	< 0.01	↑
Q6S8J3	*POTE ankyrin domain family member E*	*POTEE*	196	3.29	1.19	0.06	< 0.01	↑
P06733	*Alpha‐enolase*	*ENO1*	110	3.19	1.16	0.11	< 0.01	↑
P07737	*Profilin‐1*	*PFN1*	285	3.10	1.13	0.15	< 0.01	↑
Q562R1	*Beta‐Actin‐like protein 2*	*ACTBL2*	156	3.06	1.12	0.05	< 0.01	↑
Q8TAX7	*Mucin‐7*	*MUC7*	119	2.75	1.01	0.06	< 0.01	↑
P01034	*Cystatin‐C*	*CST3*	227	2.75	1.01	0.08	< 0.01	↑
P02810	*Salivary acidic proline‐rich phosphoprotein 1/2*	*PRH1; PRH2*	640	2.59	0.95	0.02	< 0.01	↑
P02768	*Albumin*	*ALB*	337	2.53	0.93	0.01	< 0.01	↑
P63267	*Actin, gamma‐enteric smooth muscle*	*ACTG2*	249	2.53	0.93	0.06	< 0.01	↑
P68133	*Actin, alpha skeletal muscle*	*ACTA1*	274	2.48	0.91	0.07	< 0.01	↑
P68032	*Actin, alpha cardiac muscle 1*	*ACTC1*	274	2.48	0.91	0.06	< 0.01	↑
P62736	*Actin, aortic smooth muscle*	*ACTA2*	249	2.48	0.91	0.06	< 0.01	↑
P04080	*Cystatin‐B*	*CSTB*	685	2.46	0.90	0.13	< 0.01	↑
P01876	*Immunoglobulin heavy constant alpha 1*	*IGHA1*	4648	2.44	0.89	0.02	< 0.01	↑
P04406	*Glyceraldehyde‐3‐phosphate dehydrogenase*	*GAPDH*	286	2.39	0.87	0.09	< 0.01	↑
P60709	*Actin, cytoplasmic 1*	*ACTB*	549	2.36	0.86	0.09	< 0.01	↑
P01833	*Polymeric immunoglobulin receptor*	*PIGR*	590	2.34	0.85	0.03	< 0.01	↑
P63261	*Actin, cytoplasmic 2*	*ACTG1*	544	2.27	0.82	0.05	< 0.01	↑
P0CG39	*POTE ankyrin domain family member J*	*POTEJ*	81	2.25	0.81	0.13	< 0.01	↑
A0M8Q6	*Immunoglobulin lambda constant 7*	*IGLC7*	260	2.12	0.75	0.1	< 0.01	↑
P0CF74	*Immunoglobulin lambda constant 6*	*IGLC6*	260	2.10	0.74	0.1	< 0.01	↑
P02647	*Apolipoprotein A‐I*	*APOA1*	206	2.08	0.73	0.09	< 0.01	↑
P01877	*Immunoglobulin heavy constant alpha 2*	*IGHA2*	3521	2.08	0.73	0.08	< 0.01	↑
P01591	*Immunoglobulin J chain*	*JCHAIN*	863	2.08	0.73	0.07	< 0.01	↑
P31025	*Lipocalin‐1*	*LCN1*	531	2.05	0.72	0.09	< 0.01	↑
P01023	*Alpha‐2‐macroglobulin*	*A2M*	71	2.00	0.69	0.12	< 0.01	↑
P01859	*Immunoglobulin heavy constant gamma 2*	*IGHG2*	44	2.00	0.69	0.29	0.03	↑
P02790	*Hemopexin*	*HPX*	59	1.92	0.65	0.13	< 0.01	↑
P0CG38	*POTE ankyrin domain family member I*	*POTEI*	134	1.84	0.61	0.1	< 0.01	↑
P0DOX2	*Immunoglobulin alpha‐2 heavy chain*	*IGA2*	3390	1.70	0.53	0.02	< 0.01	↑
P0DOY3	*Immunoglobulin lambda constant 3*	*IGLC3*	260	1.67	0.51	0.06	< 0.01	↑
A5A3E0	*POTE ankyrin domain family member F*	*POTEF*	196	1.67	0.51	0.07	< 0.01	↑
P61626	*Lysozyme C*	*LYZ*	650	1.67	0.51	0.04	< 0.01	↑
P0DOY2	*Immunoglobulin lambda constant 2*	*IGLC2*	260	1.63	0.49	0.05	< 0.01	↑
P0CG04	*Immunoglobulin lambda constant 1*	*IGLC1*	135	1.62	0.48	0.05	< 0.01	↑
B9A064	*Immunoglobulin lambda‐like polypeptide 5*	*IGLL5*	135	1.60	0.47	0.05	< 0.01	↑
P02787	*Serotransferrin*	*TF*	144	1.58	0.46	0.04	< 0.01	↑
P0DOX8	*Immunoglobulin lambda‐1 light chain*	*IGL1*	135	1.58	0.46	0.06	< 0.01	↑
Q8N4F0	*BPI fold‐containing family B member 2*	*BPIFB2*	251	1.42	0.35	0.06	< 0.01	↑
P09228	*Cystatin‐SA*	*CST2*	314	1.28	0.25	0.02	< 0.01	↑
P04746	*Pancreatic alpha‐amylase*	*AMY2A*	4530	1.16	0.15	0.01	< 0.01	↑
P0DUB6	*Alpha‐amylase 1A*	*AMY1A*	5510	1.15	0.14	0.01	< 0.01	↑
P19961	*Alpha‐amylase 2B*	*AMY2B*	4614	1.15	0.14	0.01	< 0.01	↑
P0DTE8	*Alpha‐amylase 1C*	*AMY1C*	5510	1.14	0.13	0.01	< 0.01	↑
P0DTE7	*Alpha‐amylase 1B*	*AMY1B*	5510	1.07	0.07	0.01	< 0.01	↑
P02814	*Submaxillary gland androgen‐regulated protein 3B*	*SMR3B*	1095	1.11	−0.10	0.02	< 0.01	↓
P01037	*Cystatin‐SN*	*CST1*	917	1.11	−0.10	0.02	< 0.01	↓
P12273	*Prolactin‐inducible protein*	*PIP*	4741	1.17	−0.16	0.03	< 0.01	↓
P01036	*Cystatin‐S*	*CST4*	2030	1.17	−0.16	0.02	< 0.01	↓
Q96DA0	*Zymogen granule protein 16 homolog B*	*ZG16B*	189	1.25	−0.22	0.05	< 0.01	↓
P01009	*Alpha‐1‐antitrypsin*	*SERPINA1*	46	1.86	−0.62	0.16	< 0.01	↓
P15516	*Histatin‐3*	*HTN3*	739	3.03	−1.11	0.08	< 0.01	↓

*Note:* Log (e) (“e” is a constant = 2.71); SD, standard deviation; p, statistical significance (adjusted by False Discovery Rate– FDR = 4); ED, Expression differences; ↑ = up‐regulated in G1 (1‐*p* > 0.95); ↓ = down‐regulated in G1 (*p* < 0.05).

**FIGURE 2:**
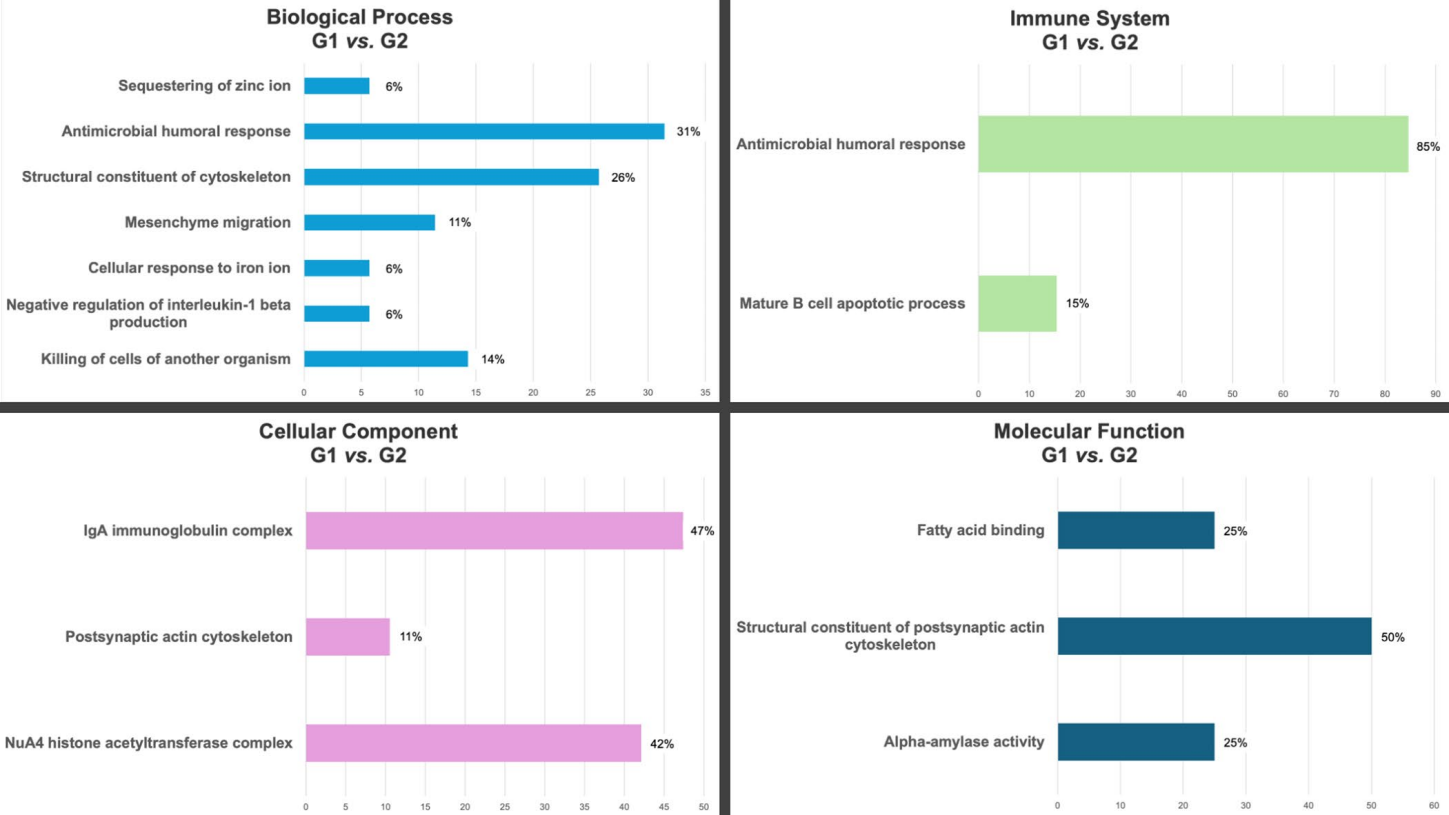
Functional analysis of differentially expressed proteins (up‐ and down‐regulated) in G1.

Based on Gene Ontology (GO) analysis, the main categories of up‐ and down‐regulated proteins in G1 were identified across the domains of biological process, immune system process, cellular component, and molecular function (Figure 2). Notably, within the Biological Process domain, the most significant categories were Antimicrobial humoral response (*p* = 1.9 × 10^−11^) and Killing of cells of another organism (*p* = 2 × 10^−4^). In the Immune System process domain, the Antimicrobial humoral response also stood out (*p* = 4.1 × 10^−7^). For the Cellular Component domain, the IgA immunoglobulin complex was the most significant category (*p* = 1.7 × 10^−21^).

**FIGURE 3:**
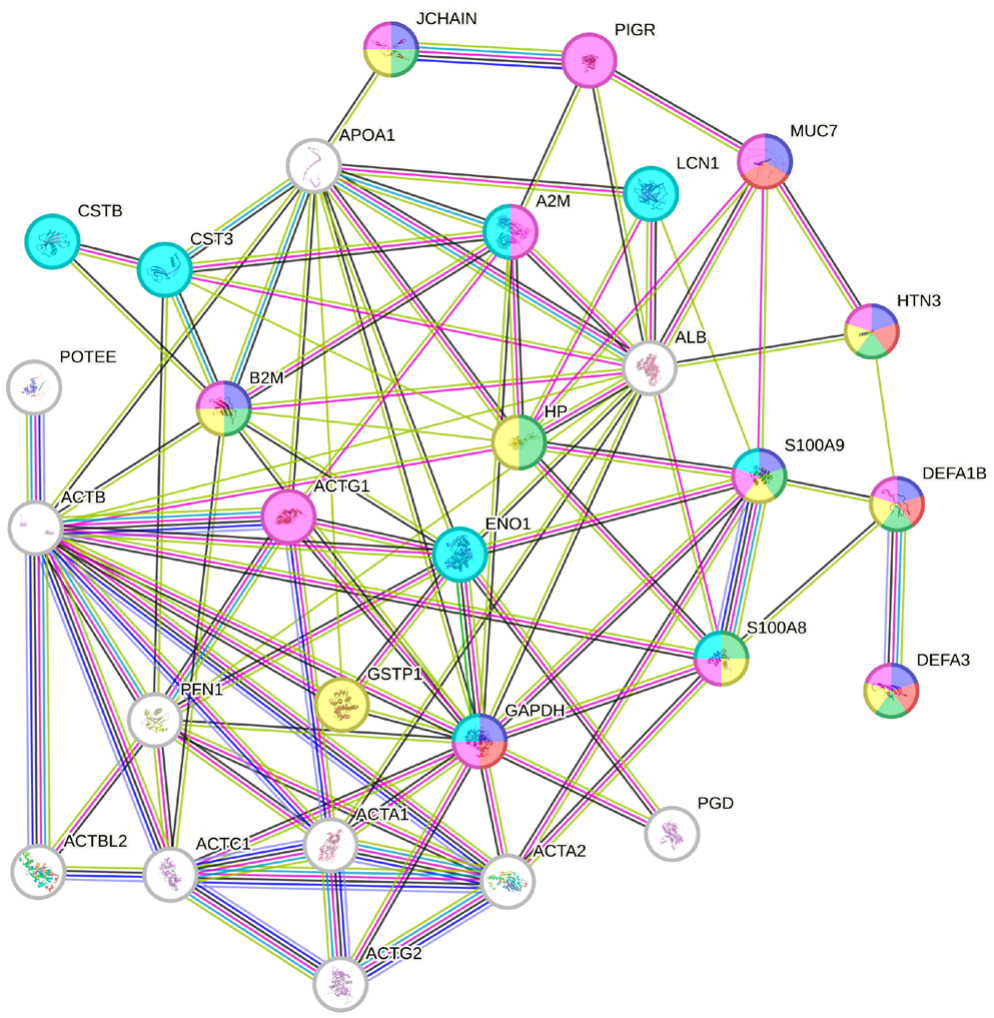
Protein–protein interaction network of up‐ and down‐regulated proteins in G1 (fold change > 2), generated in STRING. Each node represents a protein, with colors indicating involvement in specific biological processes: antimicrobial humoral response (dark blue), killing of cells of another organism (red), defense against bacteria (green), bacterial response (yellow), immune response (pink), and regulation of proteolysis (light blue). Edges depict protein–protein interactions, with colors representing the specific interaction type. Gene abbreviations correspond to proteins listed in Table 2.

STRING analysis revealed protein–protein interactions, considering only up‐ and down‐regulated proteins in G1 with fold change greater than 2 (Figure 3). The interaction analysis highlights the involvement of several proteins in key biological processes, including *antimicrobial humoral response* (dark blue nodes, FDR = 7.12 × 10^−7^), *killing of cells of another organism* (red nodes, FDR = 9.14 × 10^−5^), *defense against bacteria* (green nodes, FDR = 2.12 × 10^−5^), *bacterial response* (yellow nodes, FDR = 3.8 × 10^−4^), *immune response* (pink nodes, FDR = 2.4 × 10^−4^), and *regulation of proteolysis* (light blue nodes, FDR = 5.9 × 10^−3^) (Figure 3).

Several proteins are notable for their significant involvement in immune and antimicrobial responses, such as Protein *S100‐A9* (16‐fold increase), *Neutrophil Defensins 1* and *3* (7‐fold increase), *S100‐A8* (5‐fold increase), *Beta‐2‐Microglobulin* (4‐fold increase), *Haptoglobin* (3‐fold increase), as well as *Glyceraldehyde‐3‐Phosphate Dehydrogenase* and *Mucin‐7* (2‐fold increase). Other upregulated proteins of interest include *Alpha‐enolase* (3‐fold increase), *Cystatin‐B* and *Cystatin‐C*, *Alpha‐2‐Macroglobulin*, and *Lipocalin‐1* (2‐fold increase). Together with *S100‐A9*, *S100‐A8*, and *Glyceraldehyde‐3‐Phosphate Dehydrogenase*, these proteins play a key role in the regulation of proteolysis.

A particularly notable finding is *Histatin‐3*, the only protein reduced by more than 2‐fold (FC = 3.03). This protein plays a critical role in antimicrobial humoral response, killing cells of another organism, defense against bacteria, and immune response.

Protein validation using the MILLIPLEX multiplex immunoassay confirmed our proteomic findings, demonstrating significantly higher levels of *β‐2‐Microglobulin* and *Cystatin‐C* in G1 (Table 3).

**TABLE 3:** Protein validation using the MILLIPLEX multiplex immunoassay.

	G1 (*n* = 9) mean ± SD	G2 (*n* = 9) mean ± SD	*P*
*β‐2‐Microglobulin* (pg/ml)	121.0 ± 46.5	74.4 ± 17.0	0.015*
*Cystatin‐C* (pg/ml)	34.4 ± 6.91	26.7 ± 5.43	0.039*

Abbreviations: G1, with excessive Bleeding on Probing (BOP > 50%); G2, without generalized gingivitis (BOP < 30%); *P*, significance level; SD, standard deviation.

* *t*‐test.

## 4 Discussion

This study reveals a distinctive proteomic profile in pregnant women with severe gingivitis, supporting the hypothesis that gingival inflammation during pregnancy is associated with specific biological processes and immune responses, particularly related to antimicrobial humoral activity. Notably, some examples of the most upregulated proteins associated with excessive gingival bleeding were *Protein S100‐A9*, *Neutrophil Defensins 1* and *3*, *Protein S100‐A8*, *Beta‐2‐Microglobulin*, and *Cystatin‐C*, whereas *Histatin‐3* was the most significantly downregulated protein. These proteomic signatures were further corroborated by a validation step using a multiplex immunoassay, which confirmed higher levels of *β‐2‐Microglobulin* and *Cystatin‐C* in women with severe gingivitis. Taken together, these findings provide insights into the pathophysiological mechanisms of pregnancy gingivitis and emphasize the potential of salivary proteomics to identify candidate salivary proteins that may serve as biomarkers, pending validation in independent cohorts.

Clinically, the match of the groups concerning age, socioeconomic status, anthropometric parameters, and salivary flow ensured that differences observed in proteomic profiles were not confounded by these factors. The increased bleeding on probing (BOP) observed in G1 likely reflects the heightened inflammatory response characteristic of pregnancy‐associated gingivitis. This condition is influenced by hormonal changes, particularly elevated levels of estrogen and progesterone, which enhance vascular permeability and gingival vascularization. Additionally, the antimicrobial humoral response, as evidenced by the proteomic findings, may play a pivotal role in modulating gingival inflammation during pregnancy, contributing to the increased BOP. These mechanisms highlight the complex interplay between systemic hormonal changes and localized immune responses in the pathophysiology of pregnancy‐related gingival inflammation.

Our proteomic analysis identified 60 significantly upregulated and 7 significantly downregulated proteins in pregnant women with severe gingivitis, demonstrating distinct functional roles. Among these, *Histatin‐3* emerged as the most significantly downregulated protein, with a reduction greater than 3‐fold in pregnant women with generalized gingivitis. This antimicrobial protein plays a pivotal role in innate immune processes, particularly by protecting oral tissues from pathogens and promoting cell adhesion and migration in oral keratinocytes, gingival and dermal fibroblasts, as well as other epithelial and endothelial cells (Torres et al. 2018). Consequently, *Histatin‐3* is also a key modulator of epithelial wound healing through the facilitation of cell migration essential for tissue repair. The marked reduction in its expression observed in our study may therefore not only weaken salivary antimicrobial defenses, but also impair gingival healing capacity, favoring the persistence of inflamed areas and contributing to the chronicity of gingivitis during pregnancy.

These findings partially align with those of (Balan et al. 2021), who also reported decreased levels of salivary antimicrobial proteins in pregnant women with gingivitis, highlighting their crucial role in maintaining gingival health during pregnancy (Balan et al. 2021). However, while Balan et al. primarily emphasized reductions in salivary *Cystatins* (*S*, *SA*, and *SN*) and additional decreases in *Cystatins C* and *D*, particularly noting cystatin C's extensive involvement in major catabolic pathways and significant network re‐wiring in pregnancy gingivitis, our study revealed a contrasting pattern. In pregnancies with higher BOP, we observed decreased levels of *Cystatins‐S* and *‐SN*, alongside increased levels of *Cystatins‐B*, *‐C*, and *‐SA*.

We hypothesize that the upregulation of *Cystatins‐B*, *‐C*, and *‐SA* in G1 may represent a compensatory response to the inflammatory and microbial challenges associated with increased gingival inflammation and BOP (da Silva et al. 2023; Zemouri et al. 2019). *Cystatin‐B* may play a crucial role in mitigating proteolytic damage to gingival tissues during heightened inflammation. The increased abundance of *Cystatin‐C*, confirmed by the validation step using an immunoassay, may indicate a protective mechanism that preserves tissue integrity by modulating extracellular matrix degradation and balancing inflammatory responses (da Silva et al. 2023; Zemouri et al. 2019). Specifically, its upregulation could reflect an effort to regulate cathepsins, thereby mitigating tissue damage during enhanced inflammation. Furthermore, the elevated levels of *Cystatin‐SA*, frequently associated with salivary antimicrobial defense, could indicate an adaptive response to microbial overgrowth in pregnancy gingivitis. These findings emphasize the complexity of salivary protein dynamics in pregnancy, suggesting that *Cystatins* may have broader roles beyond antimicrobial defense, potentially encompassing tissue repair and the modulation of immune inflammatory pathways (da Silva et al. 2023; Zemouri et al. 2019).

Gene Ontology analysis provided valuable comprehensions into the biological processes associated with gingivitis in pregnancy. The most enriched categories, such as “Antimicrobial humoral response” and “Killing of cells of another organism,” indicate the intensified immune activity observed in this group. This is particularly reflected in the upregulation of key proteins, including *S100‐A9* (16‐fold increase), *Neutrophil Defensins 1* and *3* (7‐fold increase), *S100‐A8* (5‐fold increase), and *Beta‐2‐Microglobulin* (4‐fold increase). These proteins are critical mediators of antimicrobial defense and inflammatory pathways regulation. Protein–protein interaction analysis revealed that these proteins occupy central nodes in networks associated with bacterial defense, regulation of proteolysis, and immune modulation, highlighting their contribution to a robust and coordinated immune response, involving both innate and adaptive immune pathways, to maintain gingival tissues homeostasis (Balan et al. 2021; Hassan et al. 2018). For instance, the functional interaction between *Protein S100‐A9*, *Beta‐2‐Microglobulin*, and *Defensins* suggests the activation of coordinated inflammatory pathways. These findings raise the hypothesis that the regulation of these pathways may represent a potential target to modulate gingival inflammation during pregnancy.

*Proteins S100‐A9* and *S100‐A8*, members of the S100 family, are calcium‐ and zinc‐binding proteins with diverse functions, including proinflammatory, antimicrobial, oxidant‐scavenging, and apoptosis‐inducing activities. Their proinflammatory roles involve recruiting leukocytes, stimulating cytokine and chemokine production, and regulating leukocyte adhesion and migration (Grant et al. 2022; Kim et al. 2020; Wang et al. 2018). These activities are crucial for neutrophil migration to inflammatory sites, a finding that aligns closely with our observations. *Neutrophil Defensins*, essential antimicrobial peptides, enhance neutrophil‐mediated immune responses and play a pivotal role in bacterial killing (Hu and Leung 2023). In this study, we observed a remarkable sevenfold increase in *Neutrophil Defensins 1* and *3* in severe/generalized gingivitis, suggesting an enhanced role for neutrophils in pregnancy gingivitis.

During pregnancy gingivitis, neutrophils migrate to inflamed tissues, undergo degranulation, and release reactive oxygen species (ROS), which are important for bacterial clearance, but may contribute to oxidative stress (Balan et al. 2021; Nauseef 2007). The identification of neutrophil granule proteins such as *Myeloblastin* and *Myeloperoxidase* exclusively in G1 supports the presence of intensified neutrophil activity in this group. Importantly, pregnancy hormones further modulate neutrophil function: estrogens have been shown to promote proinflammatory activity, whereas progesterone can exert counterbalancing effects, mitigating excessive activation (Giaglis et al. 2016). This hormonal regulation may help explain the heightened abundance of neutrophil‐related proteins in women with severe gingivitis, reflecting not only local inflammatory changes but also systemic modulation of innate immune responses. Compensatory antioxidant mechanisms, such as the increased activity of enzymes reported by (Balan et al. 2021), are frequently observed in pregnancy gingivitis, highlighting the complex balance between oxidative stress and host defense.

In addition, *Beta‐2‐Microglobulin* (B2M) and *Alpha‐2‐Macroglobulin* (A2M) were also significantly increased in pregnancies with severe gingivitis, with the validation of the higher levels of B2M in the immunoassay. A2M is a multifunctional protein known for its role in regulating proteolytic activity during immune inflammatory responses. Its upregulation suggests a potential protective mechanism aimed at mitigating tissue damage in the context of excessive gingival inflammation (Barrera et al. 2007; Ertugrul et al. 2013). Conversely, B2M is a critical component of the major histocompatibility complex (MHC) class I molecules, playing a crucial role in antigen presentation and immune surveillance (Ertugrul et al. 2013; Tubbs et al. 2001). The increased abundance of B2M and A2M in this study reinforces their potential involvement in pregnancy gingivitis and highlights their relevance in the pathophysiology of pregnancy‐associated gingival conditions.

Although our study provides valuable insights into the proteomic changes associated with gingivitis, it is important to recognize that the scope of our analysis is less comprehensive compared to earlier published works on the topic (Bostanci et al. 2018; Bostanci et al. 2020; Zaura et al. 2020). Our proteomic approach focused on a specific set of proteins, which may not encompass the full spectrum involved in gingival inflammation. Despite this, the strengths of our analysis lie in its high sensitivity, precision, and ability to detect and quantify both individual proteins and their post‐translational modifications, crucial for understanding the inflammatory processes in gingivitis.

Other limitations should be considered. The threshold of BOP ≥ 50% adopted in this study should not be interpreted as a formal diagnostic criterion but rather as an operational strategy to characterize a subgroup with more pronounced gingival inflammation. By excluding intermediate cases (30%–50% BOP), we aimed to enhance group contrast and facilitate the identification of proteomic signatures associated with more severe gingival bleeding. Nonetheless, the relatively small sample size may limit the generalizability of our results, and the cross‐sectional design of the study prevents the establishment of causal relationships between specific proteins and gingival inflammation.

Also, although daily toothbrushing frequency did not differ significantly between groups (*p* = 0.175), this self‐reported measure is an imperfect proxy for oral hygiene. The plaque index, however, was significantly higher in G1, providing a more objective assessment of biofilm accumulation and supporting our interpretation that increased gingival inflammation was accompanied by greater biofilm levels, although residual confounding cannot be fully excluded. Furthermore, factors such as hormonal profiles and microbial loads, which were not evaluated in this study, may directly influence protein expression. Hormonal changes during pregnancy, especially increased levels of estrogen and progesterone, may enhance gingival vascular permeability and bleeding independently of plaque accumulation. This physiological condition may confound the interpretation of BOP, and our proteomic findings should therefore be interpreted as reflecting a bleeding phenotype influenced by, but not exclusively dependent on, biofilm‐driven inflammation. Future research with larger datasets, longitudinal cohorts, and a broader proteomic profiling approach should aim to validate these findings, explore additional proteins, and incorporate relevant biological factors to provide a more comprehensive understanding of the molecular mechanisms underlying gingivitis.

In conclusion, this study highlights a distinctive salivary proteomic profile in pregnant women with severe gingivitis, characterized by increased expression of inflammatory and immune‐related proteins that may contribute to the heightened gingival inflammation observed in pregnancy and provide valuable insights into the pathophysiological mechanisms underlying this condition. These proteins should be considered candidate biomarkers. Therefore, future studies, particularly longitudinal analyses in larger and independent cohorts, and involving populations with diverse contextual characteristics, are needed to confirm their predictive value and clinical applicability.

## Author Contributions

**Gerson Aparecido Foratori‐Junior:** conceptualization, investigation, funding acquisition, writing – original draft, methodology, validation, visualization, writing – review and editing, software, formal analysis, project administration, data curation, supervision, resources. **Clovis Bergamin Griso:** investigation, funding acquisition, writing – original draft, methodology, validation, visualization, writing – review and editing, resources. **Amanda Borges Pirondi:** investigation, writing – review and editing, methodology, validation, visualization, resources. **Laura Teodoro de Marchi:** investigation, writing – review and editing, methodology, validation, visualization, resources. **Talita Mendes Oliveira Ventura:** investigation, writing – review and editing, validation, methodology, visualization, software, resources, data curation. **Larissa Tercilia Grizzo:** investigation, writing – review and editing, methodology, validation, visualization, software, resources, data curation. **Roosevelt da Silva Bastos:** investigation, writing – review and editing, methodology, validation, visualization, resources. **Alejandra Chaparro:** investigation, writing – review and editing, methodology, validation, visualization, formal analysis, resources. **Nagihan Bostanci:** investigation, writing – review and editing, methodology, validation, visualization, formal analysis, resources. **Marília Afonso Rabelo Buzalaf:** investigation, funding acquisition, writing – review and editing, methodology, validation, visualization, resources, supervision.

## Ethics Statement

This study was approved by a Research Ethics Committee from Bauru School of Dentistry, University of São Paulo (CAAE 77660724.3.0000.5417). Participants were included in the study only after providing written consent.

## Conflicts of Interest

The authors declare no conflicts of interest. The authors declare that Artificial Intelligence (ChatGPT) was used, but only to improve the language of the manuscript.

## Supporting information


**Supplementary file 1** Expression differences and unique proteins identified in G1 and G2

## Data Availability

The data supporting the conclusions of this study are available from the corresponding author upon reasonable request. However, it should be noted that in addition to the availability of Supporting Information, mass spectrometry proteomic data have been deposited with the ProteomeXchange Consortium via the PRIDE partner repository (PXD059154).
