# Supplementary material for: Salivary Proteomic Signatures in Pregnant Women With Excessive Gingival Bleeding
Source: Oral Dis. 2025 Nov 2;32(3):852–63. doi: 10.1111/odi.70127 (PMC13125730; doi:10.1111/odi.70127)
Supplement: Supplementary file 1 — Expression differences and unique proteins identified in G1 and G2 [file ODI-32-852-s001.docx]

| Accession number | Protein name | Gene | Score | Fold Change | Log(e) | SD | P | ED |
| --- | --- | --- | --- | --- | --- | --- | --- | --- |
| P06702 | **Protein S100-A9** | **S100A9** | **237** | **16.44** | **2.80** | **0.02** | **< 0.01** | **↑** |
| P59665 | **Neutrophil defensin 1** | **DEFA1; DEFA1B** | **489** | **7.46** | **2.01** | **0.07** | **< 0.01** | **↑** |
| P59666 | **Neutrophil defensin 3** | **DEFA3** | **489** | **7.39** | **2.00** | **0.07** | **< 0.01** | **↑** |
| P52209 | **6-phosphogluconate dehydrogenase, decarboxylating** | **PGD** | **46** | **5.53** | **1.71** | **0.17** | **< 0.01** | **↑** |
| P01871 | **Immunoglobulin heavy constant mu** | **IGHM** | **112** | **5.31** | **1.67** | **0.08** | **< 0.01** | **↑** |
| P05109 | **Protein S100-A8** | **S100A8** | **1319** | **5.05** | **1.62** | **0.07** | **< 0.01** | **↑** |
| Q5VSP4 | **Putative lipocalin 1-like protein 1** | **LCN1P1** | **319** | **4.90** | **1.59** | **0.08** | **< 0.01** | **↑** |
| P01834 | **Immunoglobulin kappa constant** | **IGKC** | **626** | **4.81** | **1.57** | **0.03** | **< 0.01** | **↑** |
| P0DOX7 | **Immunoglobulin kappa light chain** | **IGK** | **626** | **4.71** | **1.55** | **0.1** | **< 0.01** | **↑** |
| P61769 | **Beta-2-microglobulin** | **B2M** | **182** | **4.48** | **1.50** | **0.09** | **0.01** | **↑** |
| P0DOX6 | **Immunoglobulin mu heavy chain** | **IGM** | **96** | **4.31** | **1.46** | **0.15** | **< 0.01** | **↑** |
| P0DOX5 | **Immunoglobulin gamma-1 heavy chain** | **IGG1** | **83** | **3.94** | **1.37** | **0.07** | **< 0.01** | **↑** |
| Q9BYX7 | **Putative beta-actin-like protein 3** | **POTEKP** | **94** | **3.60** | **1.28** | **0.08** | **< 0.01** | **↑** |
| P09211 | **Glutathione S-transferase P** | **GSTP1** | **72** | **3.46** | **1.24** | **0.16** | **< 0.01** | **↑** |
| P00738 | **Haptoglobin** | **HP** | **77** | **3.32** | **1.20** | **0.08** | **< 0.01** | **↑** |
| Q6S8J3 | **POTE ankyrin domain family member E** | **POTEE** | **196** | **3.29** | **1.19** | **0.06** | **< 0.01** | **↑** |
| P06733 | **Alpha-enolase** | **ENO1** | **110** | **3.19** | **1.16** | **0.11** | **< 0.01** | **↑** |
| P07737 | **Profilin-1** | **PFN1** | **285** | **3.10** | **1.13** | **0.15** | **< 0.01** | **↑** |
| Q562R1 | **Beta-actin-like protein 2** | **ACTBL2** | **156** | **3.06** | **1.12** | **0.05** | **< 0.01** | **↑** |
| Q8TAX7 | **Mucin-7** | **MUC7** | **119** | **2.75** | **1.01** | **0.06** | **< 0.01** | **↑** |
| P01034 | **Cystatin-C** | **CST3** | **227** | **2.75** | **1.01** | **0.08** | **< 0.01** | **↑** |
| P02810 | **Salivary acidic proline-rich phosphoprotein 1/2** | **PRH1; PRH2** | **640** | **2.59** | **0.95** | **0.02** | **< 0.01** | **↑** |
| P02768 | **Albumin** | **ALB** | **337** | **2.53** | **0.93** | **0.01** | **< 0.01** | **↑** |
| P63267 | **Actin, gamma-enteric smooth muscle** | **ACTG2** | **249** | **2.53** | **0.93** | **0.06** | **< 0.01** | **↑** |
| P68133 | **Actin, alpha skeletal muscle** | **ACTA1** | **274** | **2.48** | **0.91** | **0.07** | **< 0.01** | **↑** |
| P68032 | **Actin, alpha cardiac muscle 1** | **ACTC1** | **274** | **2.48** | **0.91** | **0.06** | **< 0.01** | **↑** |
| P62736 | **Actin, aortic smooth muscle** | **ACTA2** | **249** | **2.48** | **0.91** | **0.06** | **< 0.01** | **↑** |
| P04080 | **Cystatin-B** | **CSTB** | **685** | **2.46** | **0.90** | **0.13** | **< 0.01** | **↑** |
| P01876 | **Immunoglobulin heavy constant alpha 1** | **IGHA1** | **4648** | **2.44** | **0.89** | **0.02** | **< 0.01** | **↑** |
| P04406 | **Glyceraldehyde-3-phosphate dehydrogenase** | **GAPDH** | **286** | **2.39** | **0.87** | **0.09** | **< 0.01** | **↑** |
| P60709 | **Actin, cytoplasmic 1** | **ACTB** | **549** | **2.36** | **0.86** | **0.09** | **< 0.01** | **↑** |
| P01833 | **Polymeric immunoglobulin receptor** | **PIGR** | **590** | **2.34** | **0.85** | **0.03** | **< 0.01** | **↑** |
| P63261 | **Actin, cytoplasmic 2** | **ACTG1** | **544** | **2.27** | **0.82** | **0.05** | **< 0.01** | **↑** |
| P0CG39 | **POTE ankyrin domain family member J** | **POTEJ** | **81** | **2.25** | **0.81** | **0.13** | **< 0.01** | **↑** |
| A0M8Q6 | **Immunoglobulin lambda constant 7** | **IGLC7** | **260** | **2.12** | **0.75** | **0.1** | **< 0.01** | **↑** |
| P0CF74 | **Immunoglobulin lambda constant 6** | **IGLC6** | **260** | **2.10** | **0.74** | **0.1** | **< 0.01** | **↑** |
| P02647 | **Apolipoprotein A-I** | **APOA1** | **206** | **2.08** | **0.73** | **0.09** | **< 0.01** | **↑** |
| P01877 | **Immunoglobulin heavy constant alpha 2** | **IGHA2** | **3521** | **2.08** | **0.73** | **0.08** | **< 0.01** | **↑** |
| P01591 | **Immunoglobulin J chain** | **JCHAIN** | **863** | **2.08** | **0.73** | **0.07** | **< 0.01** | **↑** |
| P31025 | **Lipocalin-1** | **LCN1** | **531** | **2.05** | **0.72** | **0.09** | **< 0.01** | **↑** |
| P01023 | **Alpha-2-macroglobulin** | **A2M** | **71** | **2.00** | **0.69** | **0.12** | **< 0.01** | **↑** |
| P01859 | **Immunoglobulin heavy constant gamma 2** | **IGHG2** | **44** | **2.00** | **0.69** | **0.29** | **0.03** | **↑** |
| P02790 | Hemopexin | HPX | 59 | 1.92 | 0.65 | 0.13 | < 0.01 | ↑ |
| P0CG38 | POTE ankyrin domain family member I | POTEI | 134 | 1.84 | 0.61 | 0.1 | < 0.01 | ↑ |
| P0DOX2 | Immunoglobulin alpha-2 heavy chain | IGA2 | 3390 | 1.70 | 0.53 | 0.02 | < 0.01 | ↑ |
| P0DOY3 | Immunoglobulin lambda constant 3 | IGLC3 | 260 | 1.67 | 0.51 | 0.06 | < 0.01 | ↑ |
| A5A3E0 | POTE ankyrin domain family member F | POTEF | 196 | 1.67 | 0.51 | 0.07 | < 0.01 | ↑ |
| P61626 | Lysozyme C | LYZ | 650 | 1.67 | 0.51 | 0.04 | < 0.01 | ↑ |
| P0DOY2 | Immunoglobulin lambda constant 2 | IGLC2 | 260 | 1.63 | 0.49 | 0.05 | < 0.01 | ↑ |
| P0CG04 | Immunoglobulin lambda constant 1 | IGLC1 | 135 | 1.62 | 0.48 | 0.05 | < 0.01 | ↑ |
| B9A064 | Immunoglobulin lambda-like polypeptide 5 | IGLL5 | 135 | 1.60 | 0.47 | 0.05 | < 0.01 | ↑ |
| P02787 | Serotransferrin | TF | 144 | 1.58 | 0.46 | 0.04 | < 0.01 | ↑ |
| P0DOX8 | Immunoglobulin lambda-1 light chain | IGL1 | 135 | 1.58 | 0.46 | 0.06 | < 0.01 | ↑ |
| Q8N4F0 | BPI fold-containing family B member 2 | BPIFB2 | 251 | 1.42 | 0.35 | 0.06 | < 0.01 | ↑ |
| P09228 | Cystatin-SA | CST2 | 314 | 1.28 | 0.25 | 0.02 | < 0.01 | ↑ |
| P04746 | Pancreatic alpha-amylase | AMY2A | 4530 | 1.16 | 0.15 | 0.01 | < 0.01 | ↑ |
| P0DUB6 | Alpha-amylase 1A | AMY1A | 5510 | 1.15 | 0.14 | 0.01 | < 0.01 | ↑ |
| P19961 | Alpha-amylase 2B | AMY2B | 4614 | 1.15 | 0.14 | 0.01 | < 0.01 | ↑ |
| P0DTE8 | Alpha-amylase 1C | AMY1C | 5510 | 1.14 | 0.13 | 0.01 | < 0.01 | ↑ |
| P0DTE7 | Alpha-amylase 1B | AMY1B | 5510 | 1.07 | 0.07 | 0.01 | < 0.01 | ↑ |
| P02814 | Submaxillary gland androgen-regulated protein 3B | SMR3B | 1095 | 1.11 | -0.10 | 0.02 | < 0.01 | ↓ |
| P01037 | Cystatin-SN | CST1 | 917 | 1.11 | -0.10 | 0.02 | < 0.01 | ↓ |
| P12273 | Prolactin-inducible protein | PIP | 4741 | 1.17 | -0.16 | 0.03 | < 0.01 | ↓ |
| P01036 | Cystatin-S | CST4 | 2030 | 1.17 | -0.16 | 0.02 | < 0.01 | ↓ |
| Q96DA0 | Zymogen granule protein 16 homolog B | ZG16B | 189 | 1.25 | -0.22 | 0.05 | < 0.01 | ↓ |
| P01009 | Alpha-1-antitrypsin | SERPINA1 | 46 | 1.86 | -0.62 | 0.16 | < 0.01 | ↓ |
| P15516 | **Histatin-3** | **HTN3** | **739** | **3.03** | **-1.11** | **0.08** | **< 0.01** | **↓** |
| O75038 | 1-phosphatidylinositol 4,5-bisphosphate phosphodiesterase eta-2 | PLCH2 | 40 | G1 | G1 | G1 | G1 | G1 |
| P31947 | 14-3-3 protein sigma | SFN | 88 | G1 | G1 | G1 | G1 | G1 |
| Q01518 | Adenylyl cyclase-associated protein 1 | CAP1 | 230 | G1 | G1 | G1 | G1 | G1 |
| P52757 | Beta-chimaerin | CHN2 | 36 | G1 | G1 | G1 | G1 | G1 |
| P13929 | Beta-enolase | ENO3 | 162 | G1 | G1 | G1 | G1 | G1 |
| Q99728 | BRCA1-associated RING domain protein 1 | BARD1 | 43 | G1 | G1 | G1 | G1 | G1 |
| P00450 | Ceruloplasmin | CP | 112 | G1 | G1 | G1 | G1 | G1 |
| Q9HC52 | Chromobox protein homolog 8 | CBX8 | 41 | G1 | G1 | G1 | G1 | G1 |
| P01024 | Complement C3 | C3 | 94 | G1 | G1 | G1 | G1 | G1 |
| P35321 | Cornifin-A | SPRR1A | 850 | G1 | G1 | G1 | G1 | G1 |
| P22528 | Cornifin-B | SPRR1B | 1120 | G1 | G1 | G1 | G1 | G1 |
| P54108 | Cysteine-rich secretory protein 3 | CRISP3 | 72 | G1 | G1 | G1 | G1 | G1 |
| Q9UGM3 | Deleted in malignant brain tumors 1 protein | DMBT1 | 456 | G1 | G1 | G1 | G1 | G1 |
| Q02487 | Desmocollin-2 | DSC2 | 69 | G1 | G1 | G1 | G1 | G1 |
| P32926 | Desmoglein-3 | DSG3 | 44 | G1 | G1 | G1 | G1 | G1 |
| O96006 | E3 SUMO-protein ligase ZBED1 | ZBED1 | 31 | G1 | G1 | G1 | G1 | G1 |
| Q01469 | Fatty acid-binding protein 5 | FABP5 | 977 | G1 | G1 | G1 | G1 | G1 |
| Q6VB84 | Forkhead box protein D4-like 3 | FOXD4L3 | 85 | G1 | G1 | G1 | G1 | G1 |
| P04075 | Fructose-bisphosphate aldolase A | ALDOA | 277 | G1 | G1 | G1 | G1 | G1 |
| P09972 | Fructose-bisphosphate aldolase C | ALDOC | 132 | G1 | G1 | G1 | G1 | G1 |
| P09104 | Gamma-enolase | ENO2 | 168 | G1 | G1 | G1 | G1 | G1 |
| P06396 | Gelsolin | GSN | 45 | G1 | G1 | G1 | G1 | G1 |
| P06744 | Glucose-6-phosphate isomerase | GPI | 632 | G1 | G1 | G1 | G1 | G1 |
| P00739 | Haptoglobin-related protein | HPR | 56 | G1 | G1 | G1 | G1 | G1 |
| P34931 | Heat shock 70 kDa protein 1-like | HSPA1L | 114 | G1 | G1 | G1 | G1 | G1 |
| P0DMV8 | Heat shock 70 kDa protein 1A | HSPA1A | 127 | G1 | G1 | G1 | G1 | G1 |
| P0DMV9 | Heat shock 70 kDa protein 1B | HSPA1B | 127 | G1 | G1 | G1 | G1 | G1 |
| P11142 | Heat shock cognate 71 kDa protein | HSPA8 | 107 | G1 | G1 | G1 | G1 | G1 |
| P69905 | Hemoglobin subunit alpha | HBA1; HBA2 | 994 | G1 | G1 | G1 | G1 | G1 |
| P68871 | Hemoglobin subunit beta | HBB | 2089 | G1 | G1 | G1 | G1 | G1 |
| P02042 | Hemoglobin subunit delta | HBD | 1049 | G1 | G1 | G1 | G1 | G1 |
| P02100 | Hemoglobin subunit epsilon | HBE1 | 959 | G1 | G1 | G1 | G1 | G1 |
| P69891 | Hemoglobin subunit gamma-1 | HBG1 | 959 | G1 | G1 | G1 | G1 | G1 |
| P69892 | Hemoglobin subunit gamma-2 | HBG2 | 959 | G1 | G1 | G1 | G1 | G1 |
| P02008 | Hemoglobin subunit zeta | HBZ | 167 | G1 | G1 | G1 | G1 | G1 |
| A0A0B4J1V0 | Immunoglobulin heavy variable 3-15 | IGHV3-15 | 483 | G1 | G1 | G1 | G1 | G1 |
| A0A0B4J1Y9 | Immunoglobulin heavy variable 3-72 | IGHV3-72 | 470 | G1 | G1 | G1 | G1 | G1 |
| A0A0B4J1V6 | Immunoglobulin heavy variable 3-73 | IGHV3-73 | 470 | G1 | G1 | G1 | G1 | G1 |
| A0A075B6P5 | Immunoglobulin kappa variable 2-28 | IGKV2-28 | 401 | G1 | G1 | G1 | G1 | G1 |
| A2NJV5 | Immunoglobulin kappa variable 2-29 | IGKV2-29 | 401 | G1 | G1 | G1 | G1 | G1 |
| P06310 | Immunoglobulin kappa variable 2-30 | IGKV2-30 | 401 | G1 | G1 | G1 | G1 | G1 |
| A0A087WW87 | Immunoglobulin kappa variable 2-40 | IGKV2-40 | 401 | G1 | G1 | G1 | G1 | G1 |
| A0A0A0MRZ7 | Immunoglobulin kappa variable 2D-26 | IGKV2D-26 | 401 | G1 | G1 | G1 | G1 | G1 |
| P01615 | Immunoglobulin kappa variable 2D-28 | IGKV2D-28 | 401 | G1 | G1 | G1 | G1 | G1 |
| A0A075B6S2 | Immunoglobulin kappa variable 2D-29 | IGKV2D-29 | 401 | G1 | G1 | G1 | G1 | G1 |
| A0A075B6S6 | Immunoglobulin kappa variable 2D-30 | IGKV2D-30 | 401 | G1 | G1 | G1 | G1 | G1 |
| P01614 | Immunoglobulin kappa variable 2D-40 | IGKV2D-40 | 401 | G1 | G1 | G1 | G1 | G1 |
| P04433 | Immunoglobulin kappa variable 3-11 | IGKV3-11 | 525 | G1 | G1 | G1 | G1 | G1 |
| P01624 | Immunoglobulin kappa variable 3-15 | IGKV3-15 | 637 | G1 | G1 | G1 | G1 | G1 |
| A0A0A0MRZ8 | Immunoglobulin kappa variable 3D-11 | IGKV3D-11 | 525 | G1 | G1 | G1 | G1 | G1 |
| A0A0C4DH55 | Immunoglobulin kappa variable 3D-7 | IGKV3D-7 | 604 | G1 | G1 | G1 | G1 | G1 |
| P18510 | Interleukin-1 receptor antagonist protein | IL1RN | 157 | G1 | G1 | G1 | G1 | G1 |
| P00338 | L-lactate dehydrogenase A chain | LDHA | 423 | G1 | G1 | G1 | G1 | G1 |
| Q6ZMR3 | L-lactate dehydrogenase A-like 6A | LDHAL6A | 64 | G1 | G1 | G1 | G1 | G1 |
| P07195 | L-lactate dehydrogenase B chain | LDHB | 64 | G1 | G1 | G1 | G1 | G1 |
| P07864 | L-lactate dehydrogenase C chain | LDHC | 64 | G1 | G1 | G1 | G1 | G1 |
| P02788 | Lactotransferrin | LTF | 840 | G1 | G1 | G1 | G1 | G1 |
| Q5BKY1 | Leucine-rich repeat-containing protein 10 | LRRC10 | 86 | G1 | G1 | G1 | G1 | G1 |
| P30740 | Leukocyte elastase inhibitor | SERPINB1 | 107 | G1 | G1 | G1 | G1 | G1 |
| Q9BVV7 | Mitochondrial import inner membrane translocase subunit Tim21 | TIMM21 | 75 | G1 | G1 | G1 | G1 | G1 |
| P24158 | Myeloblastin | PRTN3 | 202 | G1 | G1 | G1 | G1 | G1 |
| P05164 | Myeloperoxidase | MPO | 74 | G1 | G1 | G1 | G1 | G1 |
| P80188 | Neutrophil gelatinase-associated lipocalin | LCN2 | 185 | G1 | G1 | G1 | G1 | G1 |
| P62937 | Peptidyl-prolyl cis-trans isomerase A | PPIA | 449 | G1 | G1 | G1 | G1 | G1 |
| Q9Y536 | Peptidyl-prolyl cis-trans isomerase A-like 4A | PPIAL4A | 92 | G1 | G1 | G1 | G1 | G1 |
| P30086 | Phosphatidylethanolamine-binding protein 1 | PEBP1 | 143 | G1 | G1 | G1 | G1 | G1 |
| P00558 | Phosphoglycerate kinase 1 | PGK1 | 225 | G1 | G1 | G1 | G1 | G1 |
| P07205 | Phosphoglycerate kinase 2 | PGK2 | 105 | G1 | G1 | G1 | G1 | G1 |
| P13796 | Plastin-2 | LCP1 | 372 | G1 | G1 | G1 | G1 | G1 |
| P13797 | Plastin-3 | PLS3 | 71 | G1 | G1 | G1 | G1 | G1 |
| A0A075B6H7 | Probable non-functional immunoglobulin kappa variable 3-7 | IGKV3-7 | 604 | G1 | G1 | G1 | G1 | G1 |
| Q16378 | Proline-rich protein 4 | PRR4 | 490 | G1 | G1 | G1 | G1 | G1 |
| Q6P5S2 | Protein LEG1 homolog | LEG1 | 225 | G1 | G1 | G1 | G1 | G1 |
| Q7Z429 | Protein lifeguard 1 | GRINA | 140 | G1 | G1 | G1 | G1 | G1 |
| P30613 | Pyruvate kinase PKLR | PKLR | 146 | G1 | G1 | G1 | G1 | G1 |
| P14618 | Pyruvate kinase PKM | PKM | 190 | G1 | G1 | G1 | G1 | G1 |
| P52566 | Rho GDP-dissociation inhibitor 2 | ARHGDIB | 348 | G1 | G1 | G1 | G1 | G1 |
| P29508 | Serpin B3 | SERPINB3 | 256 | G1 | G1 | G1 | G1 | G1 |
| P48594 | Serpin B4 | SERPINB4 | 231 | G1 | G1 | G1 | G1 | G1 |
| P35326 | Small proline-rich protein 2A | SPRR2A | 621 | G1 | G1 | G1 | G1 | G1 |
| P35325 | Small proline-rich protein 2B | SPRR2B | 417 | G1 | G1 | G1 | G1 | G1 |
| P22532 | Small proline-rich protein 2D | SPRR2D | 417 | G1 | G1 | G1 | G1 | G1 |
| P22531 | Small proline-rich protein 2E | SPRR2E | 138 | G1 | G1 | G1 | G1 | G1 |
| Q96RM1 | Small proline-rich protein 2F | SPRR2F | 138 | G1 | G1 | G1 | G1 | G1 |
| Q9BYE4 | Small proline-rich protein 2G | SPRR2G | 417 | G1 | G1 | G1 | G1 | G1 |
| Q9UBC9 | Small proline-rich protein 3 | SPRR3 | 2137 | G1 | G1 | G1 | G1 | G1 |
| Q96AH0 | SOSS complex subunit B2 | NABP1 | 84 | G1 | G1 | G1 | G1 | G1 |
| P10599 | Thioredoxin | TXN | 758 | G1 | G1 | G1 | G1 | G1 |
| Q86W42 | THO complex subunit 6 homolog | THOC6 | 136 | G1 | G1 | G1 | G1 | G1 |
| P37837 | Transaldolase | TALDO1 | 205 | G1 | G1 | G1 | G1 | G1 |
| P20061 | Transcobalamin-1 | TCN1 | 56 | G1 | G1 | G1 | G1 | G1 |
| P29401 | Transketolase | TKT | 121 | G1 | G1 | G1 | G1 | G1 |
| P60174 | Triosephosphate isomerase | TPI1 | 207 | G1 | G1 | G1 | G1 | G1 |
| P51784 | Ubiquitin carboxyl-terminal hydrolase 11 | USP11 | 57 | G1 | G1 | G1 | G1 | G1 |
| Q14508 | WAP four-disulfide core domain protein 2 | WFDC2 | 2934 | G1 | G1 | G1 | G1 | G1 |
| Q8NBB4 | Zinc finger and SCAN domain-containing protein 1 | ZSCAN1 | 74 | G1 | G1 | G1 | G1 | G1 |
| P25311 | Zinc-alpha-2-glycoprotein | AZGP1 | 126 | G1 | G1 | G1 | G1 | G1 |
| Q9H0D6 | 5'-3' exoribonuclease 2 | XRN2 | 41 | G2 | G2 | G2 | G2 | G2 |
| P24298 | Alanine aminotransferase 1 | GPT | 45 | G2 | G2 | G2 | G2 | G2 |
| P02765 | Alpha-2-HS-glycoprotein | AHSG | 89 | G2 | G2 | G2 | G2 | G2 |
| Q9GZZ8 | Extracellular glycoprotein lacritin | LACRT | 167 | G2 | G2 | G2 | G2 | G2 |
| P55075 | Fibroblast growth factor 8 | FGF8 | 66 | G2 | G2 | G2 | G2 | G2 |
| P15515 | Histatin-1 | HTN1 | 574 | G2 | G2 | G2 | G2 | G2 |
| P0DOX3 | Immunoglobulin delta heavy chain | IGD | 71 | G2 | G2 | G2 | G2 | G2 |
| P01880 | Immunoglobulin heavy constant delta | IGHD | 71 | G2 | G2 | G2 | G2 | G2 |
| P13646 | Keratin, type I cytoskeletal 13 | KRT13 | 130 | G2 | G2 | G2 | G2 | G2 |
| P19013 | Keratin, type II cytoskeletal 4 | KRT4 | 199 | G2 | G2 | G2 | G2 | G2 |
| Q14168 | MAGUK p55 subfamily member 2 | MPP2 | 47 | G2 | G2 | G2 | G2 | G2 |
| O60232 | Protein ZNRD2 | ZNRD2 | 296 | G2 | G2 | G2 | G2 | G2 |
| Q6VVX0 | Vitamin D 25-hydroxylase | CYP2R1 | 183 | G2 | G2 | G2 | G2 | G2 |
| P02774 | Vitamin D-binding protein | GC | 71 | G2 | G2 | G2 | G2 | G2 |
| P04004 | Vitronectin | GC | 41 | G2 | G2 | G2 | G2 | G2 |
| P01860 | Immunoglobulin heavy constant gamma 3 | IGHG3 | 165 | 2.05 | 0.72 | 0.36 | 0.85 | SE |
| P01861 | Immunoglobulin heavy constant gamma 4 | IGHG4 | 165 | 1.57 | 0.45 | 0.38 | 0.62 | SE |
| P23280 | Carbonic anhydrase 6 | CA6 | 813 | 1.11 | 0.10 | 0.06 | 0.94 | SE |
| P01857 | Immunoglobulin heavy constant gamma 1 | IGHG1 | 83 | 1.09 | 0.09 | 0.07 | 0.93 | SE |
| P03973 | Antileukoproteinase | SLPI | 302 | 1.08 | 0.08 | 0.13 | 0.72 | SE |
| P28325 | Cystatin-D | CST5 | 330 | 1.05 | 0.05 | 0.12 | 0.68 | SE |
| Q96DR5 | BPI fold-containing family A member 2 | BPIFA2 | 306 | 1.03 | 0.03 | 0.11 | 0.58 | SE |
| P02808 | Statherin | STATH | 2804 | 0.87 | -0.14 | 0.67 | 0.55 | SE |

Note: Log (e) (“e” is a constant = 2.71); SD, standard deviation; p, statistical significance (adjusted by False Discovery Rate - FDR = 4); ED, Expression differences; ↑ = up-regulated in G1 (1-p > 0.95); ↓ = down-regulated in G1 (p < 0.05). Bold lines indicate fold change greater than 2.
